# Phorate Exposure and Incidence of Cancer in the Agricultural Health Study

**DOI:** 10.1289/ehp.8911

**Published:** 2006-04-18

**Authors:** Rajeev Mahajan, Matthew R. Bonner, Jane A. Hoppin, Michael C.R. Alavanja

**Affiliations:** 1 Occupational and Environmental Epidemiology Branch, Division of Cancer Epidemiology and Genetics, National Cancer Institute, National Institutes of Health, Department of Health and Human Services, Rockville, Maryland, USA; 2 Epidemiology Branch, National Institute of Environmental Health Sciences, National Institutes of Health, Department of Health and Human Services, Research Triangle Park, North Carolina, USA

**Keywords:** agriculture, insecticides, neoplasms, occupational exposure, organophosphorus compounds, organothiophosphorus compounds, pesticides, prostate, phorate

## Abstract

**Background:**

We recently reported a link between use of the organophosphate pesticide phorate and risk of prostate cancer among applicators with a family history of prostate cancer in the Agricultural Health Study (AHS).

**Objective:**

This finding, together with findings of associations between other organophosphate pesticides and cancer more broadly, prompted us to examine phorate exposure and overall cancer incidence in the AHS. Adding 3 years of follow-up and using more detailed exposure information allowed us to see whether the prostate cancer finding held.

**Methods:**

The AHS is a prospective study of licensed restricted-use pesticide applicators from North Carolina and Iowa. To our knowledge, this is the largest examination of workers occupationally exposed to phorate. Pesticide exposure and other information was collected using two self-administered questionnaires completed from 1993 to 1997. Poisson regression was used to calculate rate ratios (RR) and 95% confidence intervals (CI), adjusting for potential confounders.

**Results:**

Phorate use was not related to the incidence of all cancers combined or to any individual cancer, although we had insufficient numbers to study non-Hodgkin lymphoma or leukemia, which have been linked to organophosphates in other studies. Although prostate cancer risk was not significantly related to phorate use overall or among those without a family history, the risk tended to increase among applicators with a family history of prostate cancer. The interaction RR was 1.53 (95% CI, 0.99–2.37).

**Conclusion:**

The observed statistical interaction suggests a gene–environment interaction between family history and phorate exposure in the incidence of prostate cancer, but other explanations are also possible.

Phorate {*O*,*O*-diethyl *S*-[(ethylthio)methyl] phosphorothioate; trade name: thimet} is an organophosphate compound used agriculturally, primarily in the production of corn, cotton, and potatoes, to control sap-feeding insects including various beetles, mites, grubs, and worms. There are no registered residential uses. Phorate was first registered for use in the United States in 1959 [U.S. Environmental Protection Agency (EPA) 2001]. In the United States, almost 2.5 million acres are treated annually with 2–3 million pounds of phorate, making it the sixth most common organophosphate used (Donaldson et al. 2002).

The currently limited body of literature does not provide evidence to suggest that phorate is mutagenic, genotoxic, or carcinogenic (Bingham et al. 2001; California Department of Pesticide Regulation 1996; Lin et al. 1987; Pandita 1986). However, several epidemiologic studies have found associations between exposure to organophosphate pesticides and non-Hodgkin lymphoma (NHL) (Cantor et al. 1992; Zahm et al. 1993) as well as leukemia (Brown et al. 1990; Clavel et al. 1996), and the International Agency for Research on Cancer (IARC) considers insecticide application to be an exposure that is probably carcinogenic in humans (Group 2A) (IARC 1991). Recent findings from the Agricultural Health Study (AHS) linking lung cancer with exposure to diazinon and chlorpyrifos (Alavanja et al. 2004) and prostate cancer with exposure to chlorpyrifos, coumaphos, fonofos, and phorate among applicators with a family history of prostate cancer (Alavanja et al. 2003) prompted us to examine risk for all cancers among phorate users in the same cohort over a longer follow-up period. The aforementioned insecticides belong to the organothio-phosphate subgroup, are similar to phorate in chemical structure, and must be converted in the body to their bioactive, neurotoxic oxon forms, which irreversibly inhibit acetylcholine esterase by phosphorylating a serine hydroxyl group in the active site of the enzyme (Pope 1999; Sultatos 1994). Little is known of the carcinogenicity of the oxon species. To our knowledge, this is the largest epidemiologic examination of an occupational group exposed to phorate.

## Methods

### Cohort enrollment and follow-up

The AHS has been described elsewhere (Alavanja et al. 1996). It is a prospective cohort including 52,395 private applicators (farmers) from Iowa and North Carolina and 4,916 commercial applicators (employees of pest control companies or persons who apply pesticides as employees of businesses whose primary function is not pesticide application) from Iowa licensed to apply restricted-use pesticides (82.4% of eligible applicators). Incident tumors diagnosed between enrollment (31 December 1993 to 31 December 1997) and 31 December 2002 were identified using population-based tumor registries of both states. Subjects were censored in the year they moved out of the state, as determined by an extensive search of address records, or the year they died, as determined using the National Death Index and state death registry records. Less than 2% of the cohort was lost to follow-up. The average follow-up time of 7.5 years represents an increase of 3.2 years over the previously published prostate cancer paper. All participants provided informed consent and the protocol was approved by all appropriate institutional review boards.

### Exposure assessment

Applicators were given two self-administered questionnaires upon enrollment. The enrollment questionnaire collected information on days of use per year, years of use, and decade of first use for 22 pesticides, as well as information on ever/never use of 28 additional pesticides (including phorate); application methods; use of personal protective equipment (PPE); smoking; alcohol consumption; farm activities; cancer history in first-degree relatives; and basic demographic data. A supplemental take-home questionnaire collected information on days of use per year, years of use, and decade of first use for the 28 pesticides for which only ever/never use information was collected in the enrollment questionnaire. The take-home questionnaire, which was completed by 25,291 (44%) of the participants, also collected additional information on work practices, physical activity, medical conditions, and occupational exposures from nonfarming jobs. Both questionnaires are available at http://www.aghealth.org/questionnaires.html. Applicators who did not return a questionnaire were generally similar to those who did with respect to many characteristics including use of crop insecticides; however, small differences were observed with respect to education, age, family size, and vegetable consumption (Tarone et al. 1997). Because of changing pesticide exposure patterns over time, pesticide exposure information, pesticide handling practices, and other information was updated by computer-aided telephone interview from 1999 to 2003. However, because of the proximity of this interview period to the end of cancer incidence follow-up, it is unlikely that this exposure information was etiologically relevant, and we have not used the interview information here.

We estimated phorate exposure using phorate lifetime exposure-days, calculated by multiplying the frequency of phorate use in an average year and the number of years of use, using the midpoints of the questionnaire categories, and intensity-weighted exposure-days, calculated by multiplying lifetime exposure-days by an intensity score calculated using the following algorithm: intensity score = (mixing status + application status + equipment repair) × PPE (Dosemeci et al. 2002). This algorithm takes into account the effect of exposure-modifying factors by assigning different weights to various activities based on the inverse of their potential contribution to total exposure. For example, because dermal absorption is often the most important exposure route for pesticide applicators (Maroni et al. 2000), the use of chemically resistant gloves was weighted to confer a greater reduction in intensity score than any other single item of PPE; the use of disposable outer clothing reduced the intensity score to a lesser degree.

### Statistical analysis

In contrast to the previously published tumor-specific analysis of prostate cancer, which examined prostate cancer risk with respect to the ever/never use of pesticides among all male pesticide applicators without a prior prostate cancer diagnosis, this analysis was limited to the subset of applicators who completed the take-home questionnaire. Prevalent cancer cases (*n* = 620) and applicators who did not provide information on phorate exposure or other variables (*n* = 3,655) were excluded from this analysis, leaving 5,903 exposed and 15,113 nonexposed applicators. Participants with missing information tended to be older and to live in North Carolina. They were also likely to have missing information for more than one of the variables listed above.

Cancer sites were selected for analysis if there were 15 or more incident diagnoses among phorate-exposed subjects. Specifically, these sites were *a*) all cancers combined; *b*) cancers of the colon, lung, and prostate; and *c*) the grouping of lymphohematopoietic cancers, which contains both Hodgkin lymphoma and NHL, leukemia, and multiple myeloma. All statistical analyses were conducted in AHS data release 0412.01 using Stata version 8 (StataCorp, College Station, TX; StataCorp 2003). Poisson regression was used to calculate incidence rate ratios (RR) and 95% confidence intervals (CI). Both lifetime exposure-days and intensity-weighted exposure-days for exposed applicators were categorized into tertiles based on the distribution of the exposure metric among cancer cases. To improve resolution at high exposure levels, we further categorized lifetime exposure-days by splitting the top tertile at the median when the split left at least four exposed cases in each new category. With intensity-weighted exposure-days used as the exposure measurement, the highest tertile was not split because of a small number of cases. Finally, because measures of lifetime cumulative exposure do not distinguish between infrequent exposure over a long period of time and more frequent exposure over a short period of time, which could make a difference with respect to cancer, we examined cancer incidence in relation to average days of use per year categorized as none, low, and high (low and high categorized at the median) and stratified by low and high years of use (categorized at median).

Both the lifetime and intensity-weighted exposure-days analyses used the nonexposed and lowest exposed categories as the reference group. Cancer-specific analyses were adjusted for age as a continuous variable; applicator type (private or commercial); state of residence (Iowa or North Carolina); education (≤ high school graduate, > high school graduate); pack-years of smoking categorized at the median (never, ≤ 12, > 12); history of the specific tumor in first-degree relatives (yes or no); and use of the five most correlated pesticides [aldicarb, ethylene dibromide, aldrin, 2,4,5-trichlorophenoxy propionic acid (2,4,5-TP), and butylate]. Pearson correlation coefficients ranged from 0.39 (aldicarb) to 0.36 (butylate). We categorized use of each correlated pesticide as never, low, and high usage, employing the median lifetime exposure-days of use to distinguish between low and high usage. Although the results of these more fully adjusted analyses and the analyses adjusted for age and smoking only were consistent, we present the more fully adjusted results to address the fact that farmers are exposed to many agents. To assess trends in dose–response patterns, we performed linear trend tests by assigning each exposure category the median value in that category and treating the variable as a continuous variable.

To further examine the risk of prostate cancer, we obtained a parsimonious model by removing variables that did not alter point estimates by > 5%, leaving variables for age and state of residence in the model. We used an interaction term obtained by taking the product of family history of prostate cancer and category of lifetime exposure-days to evaluate effect modification between phorate exposure and family history of prostate cancer.

## Results

Table 1 displays selected characteristics of applicators by their level of exposure, with “lowest exposed” referring to those in the lowest exposed tertile of lifetime exposure-days, and “other exposed” referring to those in the remaining tertiles. Overall, those in the nonexposed category tended to be less likely to report producing corn, slightly less likely to report family history of any cancer, and younger than those in either of the exposed categories. Additionally, compared with the exposed applicators, the nonexposed were also exposed to significantly fewer of the pesticides that were assessed in the questionnaires. Finally, residents of North Carolina were more likely than residents of Iowa to be nonexposed. These differences between the nonexposed group and either of the two exposed groups suggest that the lowest exposed group may be the more appropriate reference group.

Table 2 displays adjusted associations between selected cancer sites and phorate lifetime exposure-days. Phorate use did not appear to be associated with the incidence of all cancers combined. For prostate and colon cancer, the results differed depending on the reference group. The risk estimates for both cancers increased monotonically with increasing exposure category relative to the lowest exposed, but the point estimates and linear trend tests were not significant. However, the point estimates were not elevated compared with the nonexposed. Phorate use was not related to any other examined cancer.

Because phorate use was uncommon in North Carolina, we repeated our analysis of all cancers combined, restricting the data to applicators from Iowa. The results were similar to those presented above (not shown). They were not meaningfully different when we adjusted for lifetime exposure-days to all pesticides rather than the five most correlated pesticides (not shown). Finally, the results were also consistent when we examined days of use per year by years of use (not shown). Namely, we observed slightly elevated but insignificant point estimates for prostate and colon cancer, and no discernible pattern with any other examined cancer.

Because of the large number of applicators excluded due to missing information on covariates, we repeated the analysis in Table 2 on all applicators without missing phorate exposure information by assigning the missing covariates an unspecified category (not shown). The results of this analysis of 17,051 nonexposed and 6,488 exposed applicators did not substantially differ from the results presented above.

Using the intensity-weighted exposure-days metric, though the number of cases was slightly reduced because individuals were missing data on intensity metric covariates, except for prostate cancer the results were not meaningfully different from those presented above (not shown). Phorate use was not associated with all cancers combined. Colon cancer risk estimates were not elevated using the nonexposed reference group, but compared with the lowest exposed, they increased monotonically with exposure category (RR in highest exposed = 1.81; 95% CI, 0.58–5.60). RRs for prostate cancer were uninfluenced by exposure category regardless of reference group. Phorate use was not associated with any other examined cancers.

Although too few exposed melanoma cases (*n* = 14) prevented inclusion of results in tables, there were some interesting yet statistically insignificant observations. In particular, melanoma risk estimates tended to increase with lifetime and intensity-weighted exposure-days category regardless of reference group. Relative to the unexposed, risk estimates were also elevated among low and high days of use per year, but only in applicators with many years of use.

When prostate cancer risk was stratified by family history of prostate cancer, risk estimates did not increase across categories of lifetime exposure-days in the stratum of applicators reporting no family history (Table 3). However, in applicators with a family history, the risk estimates tended to increase. The RR in the highest compared to the lowest exposed was 1.91 (95% CI, 0.86–4.24). The interaction RR indicated that the risk associated with an increase in phorate exposure category was 1.53 (95% CI, 0.99–2.37) times higher in those with a family history of prostate cancer compared with those without. To account for latency of exposure, we repeated the analysis among applicators whose first use of phorate was prior to 1980 (not shown). Too few exposed applicators with a family history of prostate cancer prevented us from conducting the same analysis among those whose first exposure was after 1980. The results were consistent with those presented above.

For comparison with the previously published prostate cancer analysis (Alavanja et al. 2003), we repeated the analysis using methodology comparable to that used in the previously published prostate cancer study. That is, we used logistic regression to calculate odds ratios (OR) among all enrolled male applicators without prior history of prostate cancer, categorizing phorate exposure as ever/never. We used the cross product of ever/never phorated exposure and family history of prostate cancer to assess effect modification (not shown). We found that the age-adjusted prostate cancer risk was significantly elevated in those with a family history of prostate cancer (OR = 1.53; 95% CI, 1.09–2.14; 249 exposed cases), but not in those without (OR = 1.11; 95% CI, 0.95–1.31; 73 exposed cases). The interaction OR was 1.40 (95% CI, 0.96–2.04), adjusted for age and family history of prostate cancer. The corresponding interaction OR from the previous paper was 1.64 (95% CI, 1.02–2.63).

## Discussion

Phorate use was not related to the occurrence of all cancers combined in this study. Although previous studies have observed suggestive increases in the risk of NHL and leukemia associated with the use of organophosphate pesticides (Brown et al. 1990; Cantor et al. 1992; Clavel et al. 1996; Zahm et al. 1993), the risk of lymphohematopoietic cancers overall was not associated with phorate use in this cohort. Too few exposed cases of NHL, leukemia, Hodgkin lymphoma, and multiple myeloma prevented evaluations of these cancers individually. As the lymphohematopoietic grouping may not be etiologically homogeneous, it would be prudent for follow-up studies to examine each cancer separately as more cancer cases develop in the cohort.

Prostate cancer risk was not significantly associated with phorate use. However, among applicators reporting a family history of prostate cancer, the risk associated with phorate exposure was elevated, whereas there was no corresponding increase among those without a family history. An elevated interaction term of similar magnitude was observed in an examination of prostate cancer in an article by Alavanja et al. (2003). The study conducted here is a chemical-specific analysis carried out on 135 phorate-exposed prostate cancer cases using information on lifetime exposure-days and intensity-weighted lifetime-exposure days to examine dose–response relationships. In contrast, the previously published prostate cancer study was a tumor-specific analysis and in quantifying phorate use as ever/never use could not examine dose–response trends. In addition, this study was carried out over a longer average follow-up period of 7.5 years, compared with 4.3 years. Despite the analytic differences, the results are generally consistent with the previous paper.

Family history of prostate cancer is strongly and consistently linked to prostate cancer in the scientific literature (Bostwick et al. 2004). For example, monozygotic twins have higher prostate cancer concordance than dizygotic twins (Gronberg et al. 1994). Risk is elevated several-fold in individuals with an affected father or brother (Glover et al. 1998; Spitz et al. 1991; Steinberg et al. 1990; Whittemore et al. 1995) and may increase if a greater number of first-degree relatives are affected (Steinberg et al. 1990). Finally, cancers in individuals with affected family members occur at younger ages compared with individuals without affected family members (Bratt et al. 1999; Gronberg et al. 1999). Farming occupation has also been modestly but significantly associated with prostate cancer (Blair et al. 1985; Checkoway et al. 1987; Sharma-Wagner et al. 2000; van der Gulden and Vogelzang 1996; Van Maele-Fabry and Willems 2004). In particular, the results of several studies suggest that this association may be caused by exposure to pesticides (Potti et al. 2003). Significant associations were found specifically among those individuals who were ever employed in the mixing and application of pesticides (Fleming et al. 1999; Settimi et al. 2001), and cancer risk increased with both the number of days of pesticides applied per year (van der Gulden et al. 1995) and the number of acres sprayed with herbicides in 1 year (Morrison et al. 1993).

Although a number of exposures shared in common between study subjects and their first-degree relatives could lead to a statistical interaction between phorate use and family history of prostate cancer, the presence of family history of prostate cancer may serve as a surrogate for an inherited genetic trait, such as a polymorphism in a metabolism enzyme. The active form of most organophosphates, including phorate and chlorpyrifos, is the corresponding oxon (Pope 1999; Sultatos 1994), and both of these insecticides are metabolized using many of the same enzymes (Tang et al. 2001; Usmani et al. 2004). Polymorphic variants of several cytochrome P450 isoforms vary considerably in their ratio of bioactivation to detoxification of chlorpyrifos (Dai et al. 2001; Tang et al. 2001). Thus, it is possible that the observation of an interaction of family history and phorate exposure reflects the presence of an inherited polymorphism that alters the balance between bioactivation and detoxification in the body.

There are some limitations of this study. At this time, investigation of certain cancers is hindered by small numbers of exposed cases, making it difficult to analyze some cancers. However, as the cohort ages, more cancer cases will accrue and allow for more statistically powerful investigations. Additionally, some exposure misclassification is likely, although there is no reason to believe that it occurred differentially between cancer cases and cancer-free subjects, because exposure information was gathered prior to disease onset.

Another general limitation of studies of pesticide applicators is that few applicators are exposed to one agent. To attempt to control for potential confounding from other pesticides, the risk estimates were adjusted for use of the five pesticides most correlated with phorate. However, the use of other pesticides did not likely confound the observed relationships because the correlation coefficients, which ranged between 0.36 for butylate and 0.39 for aldicarb, were not very high. Moreover, risk estimates were similar when they were adjusted for cumulative lifetime exposure-days to all pesticides. An examination of pesticide usage and specific farming activities found that these activities likely resulted in minimal confounding (Coble et al. 2002).

The exposure measures in this study are an improvement. Whereas previous studies of pesticide exposures were limited to qualitative exposure measures, this study attempts to quantify cumulative lifetime exposure by incorporating measures of frequency, duration, and intensity of exposure to specific pesticides. The measure of intensity used information such as application methods and PPE use to calculate a more precise measurement of actual exposure. Furthermore, the rate of recruitment and follow-up of participants was very high, as 82% of eligible participants enrolled, and < 2% were lost to follow-up. Although not all take-home questionnaires were returned, the measured differences between respondents and nonrespondents were not likely to be influential here (Tarone et al. 1997). Thus, the applicators returning the take-home questionnaire were likely representative of the overall cohort in terms of cancer risk.

In summary, no clear association between phorate and any cancer was observed in this study. However, the study findings are not inconsistent with the hypothesis that there is an interaction between phorate exposure and family history of prostate cancer in the incidence of this cancer, suggesting that there may be an inherited genetic variability that alters susceptibility to prostate cancer. Future analyses of the AHS to further investigate the relationship between phorate exposure and the risk of prostate cancer are warranted.

## Figures and Tables

**Table 1:** Characteristics of applicators by phorate exposure in the AHS (1993–1997) [no. (%)].

Characteristic	Nonexposed *n* = 15,113	Lowest exposed *n* = 2,407	Other exposed *n* = 3,496
Age (years)			
< 40	1,981 (13.1)	167 (6.9)	218 (6.2)
40–49	4,161 (27.5)	581 (24.1)	797 (22.8)
50–59	3,630 (24.0)	666 (27.7)	992 (28.4)
≥ 60	5,341 (35.3)	993 (41.3)	1,489 (42.6)
Sex			
Male	14,589 (96.5)	2,387 (99.2)	3,475 (99.4)
Female	524 (3.5)	20 (0.8)	21 (0.6)
State of residence			
Iowa	9,783 (64.7)	2,261 (93.9)	2,945 (84.2)
North Carolina	5,330 (35.3)	146 (6.1)	551 (15.8)
Applicator type			
Commercial	1,779 (11.8)	101 (4.2)	219 (6.3)
Private	13,334 (88.2)	2,306 (95.8)	3,277 (93.7)
Smoking history			
Never	8,262 (54.7)	1,429 (59.4)	1,977 (56.6)
Light (< 12 pack-years)	3,437 (22.7)	542 (22.5)	793 (22.7)
High (≥ 12 pack-years)	3,414 (22.6)	436 (18.1)	726 (20.8)
Education			
≤ High school	8,183 (54.2)	1,273 (52.9)	2,050 (58.6)
> High school	6,930 (45.9)	1,134 (47.1)	1,446 (41.4)
Family history of cancer^a^			
No	8,257 (58.7)	1,225 (53.0)	1,763 (53.0)
Yes	5,809 (41.3)	1,084 (47.0)	1,561 (47.0)
Alcohol^a^,^b^			
No	5,149 (34.8)	593 (24.9)	944 (27.4)
Yes	9,641 (65.2)	1,789 (75.1)	2,506 (72.6)
Corn farming			
No	4,897 (32.4)	149 (6.2)	366 (10.5)
Yes	10,216 (67.6)	2,258 (93.8)	3,130 (89.5)
Use of correlated pesticides			
Aldicarb	932 (6.2)	111 (4.6)	444 (12.7)
Ethylene dibromide	687 (4.6)	42 (1.8)	102 (2.9)
Aldrin	1,466 (9.7)	674 (28.0)	1,028 (29.4)
2,4,5-TP	590 (3.9)	122 (5.1)	257 (7.4)
Butylate	3,038 (20.1)	940 (39.1)	1,437 (41.1)
No. of pesticides used^c^	10.7 ± 5.9	16.0 ± 5.9	17.0 ± 6.5

a Column numbers do not add up to total *n* because of missing information.

b Based on reported alcohol consumption in the past 12 months.

c Mean ± SD.

**Table 2:** RRs (95% CIs) for selected cancers by phorate lifetime exposure-days among AHS (1993–1997) applicators, using nonexposed and lowest exposed applicators as the reference group.

Lifetime exposure-days^a^	Cases (*n*)	Nonexposed reference	Lowest exposed reference
All cancer			
0	689	1.00	
> 0–8.75	111	0.84 (0.68–1.04)	1.00
> 8.75–38.75	84	0.94 (0.74–1.19)	1.14 (0.85–1.53)
> 38.75–108.5	62	0.89 (0.68–1.17)	1.08 (0.78–1.49)
> 108.5	38	0.94 (0.67–1.33)	1.19 (0.79–1.78)
*p-*Trend		0.71	0.53
Lymphohematopoietic cancer			
0	72	1.00	
> 0–8.75	9	0.59 (0.20–1.12)	1.00
> 8.75–38.75	9	0.88 (0.40–2.03)	1.24 (0.48–3.22)
> 38.75	8	0.64 (0.52–2.17)	0.84 (0.30–2.33)
*p-*Trend		0.30	0.57
Colon cancer			
0	53	1.00	
> 0–8.75	6	0.47 (0.20–1.13)	1.00
> 8.75–38.75	7	0.90 (0.40–2.03)	2.22 (0.72–6.83)
> 38.75	10	1.07 (0.49–2.52)	2.48 (0.84–7.36)
*p-*Trend		0.74	0.18
Lung cancer			
0	69	1.00	
> 0–8.75	6	0.81 (0.34–1.96)	1.00
> 8.75–38.75	5	1.00 (0.39–2.61)	1.14 (0.34–3.84)
> 38.75–108.5	4	0.82 (0.29–2.37)	0.85 (0.22–3.23)
> 108.5	4	0.95 (0.31–2.94)	0.63 (0.14–2.86)
*p-*Trend		0.91	0.47
Prostate cancer			
0	286	1.00	
> 0–8.75	53	0.89 (0.65–1.21)	1.00
> 8.75–38.75	38	0.91 (0.64–1.29)	1.08 (0.71–1.66)
> 38.75–108.5	28	0.92 (0.62–1.38)	1.16 (0.73–1.86)
> 108.5	16	0.93 (0.55–1.57)	1.31 (0.72–2.37)
*p-*Trend		0.78	0.40

*p*-Trend, *p*-value for trend test. Incidence RRs adjusted for age, state of residence, applicator type, education, family history of site-specific cancer, smoking, and use of aldicarb, ethylene dibromide, aldrin, 2,4,5-TP, and butylate.

a Lifetime exposure-days = years of use × days of use per year.

**Table 3:** Incidence RRs^a^ (95% CIs) for prostate cancer by phorate lifetime exposure-days after stratification by family history of prostate cancer among male AHS (1993–1997) applicators.

Lifetime exposure-days^b^	Cases (*n*)	Nonexposed reference	Lowest exposed reference
No family history			
0	270	1.00	
> 0–8.75	49	1.01 (0.74–1.39)	1.00
> 8.75–24.5	35	1.05 (0.73–1.51)	1.03 (0.67–1.60)
> 24.5	35	0.91 (0.64–1.30)	0.92 (0.59–1.43)
*p-*Trend		0.66	0.69
Family history			
0	56	1.00	
> 0–8.75	10	0.69 (0.35–1.39)	1.00
> 8.75–24.5	11	1.27 (0.65–2.49)	1.90 (0.80–4.50)
> 24.5	17	1.48 (0.85–2.58)	1.91 (0.86–4.24)
*p-*Trend		0.11	0.16
Interaction^c^		1.18 (0.96–1.44)	1.53 (0.99–2.37)

*p*-Trend, *p*-value for trend test.

a RRs adjusted for age and state of residence.

b Lifetime exposure-days = years of use × days of use per year.

c RR and 95% CI for the cross product of family history of prostate cancer and lifetime exposure-days category, adjusted for age, state of residence, and family history of prostate cancer.
